# 

**Published:** 1889-07

**Authors:** 


THE PATHOLOGY OF ACTINOMYCOSIS, WITH RECORD OF CASES AND EXPERIMENTS. George A. Bodamer, M.D., B.S. . . . . . 195
THE VENA AZYGOS IN CLOVEN FOOTED ANIMALS. R. W. Burke, M.R.C.V.S . . . . . . 210
SOME OBSERVATIONS ON COCCIDIA IN THE RENAL EPITHELIUM OF THE MOUSE. Theobald Smith, M.D . . . . . . 211
OSTEOLOGICAL STUDIES OF THE SUB-FAMILY ARDEINÆ. R. W. Shufeldt, M.D., C.M.Z.S. 218
CLINICAL AND PATHOLOGICAL NOTES FROM A BREEDING STATION. Wesley Mills, M.A., M.D. . . . . . . 243
LECTURE ON EMERGENCIES OF THE HORSE AND THEIR TREATMENT. Major R. S. Huidekoper. . . . . . . 247
EDITORIAL DEPARTMENT . . . . . . 263
CASE DEPARTMENT . . . . . . 269
SELECTIONS FROM FOREIGN JOURNALS . . . . . . 273
PROCEEDINGS OF VETERINARY SOCIETIES . . . . . . 275
REVIEW DEPARTMENT . . . . . . 282
BOOKS AND PAMPHLETS RECEIVED . . . . . . 283

